# 

**Published:** 1888-09

**Authors:** 


### CONTENTS:

ARTICLE I.—Some Affections of the Gums.
By Frank Lankester, England .......... 193
" II.—Pyorrhœa Alveolaris, Etiology and Treatment.
By J. D. Patterson, D. D. S .......... 205
" III.—Ulcerative Diseases of the Mouth.
By George J. Dennis, M. D., D. D. S., St. Louis, Mo... 216
" IV.—Labial and Palatine Cavities—Cause, Mode of Preparation, and With Whot to Fill Them. By H. F. Harvey, D. D. S., Cleveland, O .......... 223
" V.—The Sixth Year Molar. By W. H. Hall, D. D. S .......... 229

### EDITORIAL.

Prosecution of American Dentists .......... 232
A Double Tragedy .......... 235
A Doctor's Narrow Escape .......... 237

### OBITUARY.

Dr. George W. Keely .......... 238

### MONTHLY SUMMARY.

Erythrophlæine as a Local Anæsthetic .......... 239

Epistaxis: Lemon Juice .......... 240

Treatment of Burns .......... 240

